# Severe Electrolyte Abnormalities and Distal Renal Tubular Acidosis in the Setting of Apremilast Use for Psoriatic Arthritis: A Case Report

**DOI:** 10.7759/cureus.53514

**Published:** 2024-02-03

**Authors:** Brody M Fogleman, Emilie McKinnon, Schuyler Nebeker, Kedareeshwar S Arukala

**Affiliations:** 1 Internal Medicine, Edward Via College of Osteopathic Medicine, Spartanburg, USA; 2 Internal Medicine, Grand Strand Medical Center, Myrtle Beach, USA

**Keywords:** electrolyte disturbance, hypokalemia, apremilast, psoriatic arthritis, renal tubular acidosis

## Abstract

Renal tubular acidosis (RTA) involves dysfunction of the renal tubular system, which leads to electrolyte abnormalities and acid-base dysregulation. The case we present here discusses a patient with a past medical history of psoriatic arthritis who presented to the emergency department with progressive generalized weakness and anorexia in the preceding four weeks. She was found to have profound hypokalemia (1.2 mmol/L), hyperchloremic metabolic acidosis, and multiple other electrolyte abnormalities. Following an extensive workup, her principle problem was deemed to be distal (type 1) RTA. She was treated with sodium bicarbonate, spironolactone, and aggressive rehydration, which eventually led to the stabilization of her electrolytes alongside clinical improvement over the course of an eight-day hospitalization. The workup did not reveal a clear etiology for the RTA. One month prior to hospitalization, she was started on apremilast, a new medication for her psoriatic arthritis. Given the limited availability of alternative explanations and the temporality of clinical manifestations, our findings raise suspicion that apremilast might be associated with her clinical presentation.

## Introduction

The renal tubular system of the nephron plays a crucial role in the maintenance of electrolytes, acid-base, and fluid balance. Renal tubular acidosis (RTA) is characterized by the inability of the kidneys to adequately maintain normal serum electrolytes due to tubular dysfunction [1]. RTA encompasses a diverse range of conditions in which metabolic acidosis occurs because of abnormalities in the renal tubules responsible for maintaining acid-base balance [2]. In these cases, however, the glomerular filtration rate remains relatively intact [2]. Type 1 RTA, also known as distal RTA, is one of the three main types of RTAs [1]. It results from a compromised ability to acidify urine, often due to reduced H+ secretion via the Na+/H+-ATPase in the distal nephron [1], resulting in potassium wasting and hypokalemia [3,4]. While autoimmune diseases elevate the risk of distal RTA, Sjögren’s Syndrome is the most commonly associated condition [5]. However, there is limited evidence supporting psoriatic arthritis as a possible cause. Apremilast, a treatment for psoriatic arthritis that inhibits phosphodiesterase-4 and modulates downstream pro-inflammatory signaling [6], has been linked to proximal RTA in a single case study [7]. Other known causes of distal RTA include systemic lupus erythematosus [5], nephrolithiasis, cirrhosis, and nephrotoxic agents such as amphotericin B, lithium, and foscarnet [8].

## Case presentation

A 73-year-old female with a past medical history of psoriatic arthritis, osteoporosis, hypertension, hyperlipidemia, and gastroesophageal reflux disease presented with a four-week history of generalized weakness, severe anorexia, and falls. A review of systems was notable for an unintentional 18.1 kg (40 lb) weight loss, confusion, nausea, vomiting, and severe constipation. The patient was undergoing outpatient workup for anemia and leukocytosis. She had recently completed three iron transfusions and a bone marrow biopsy, which showed no acute abnormalities. Home medications consisted of daily 12.5 mg hydrochlorothiazide, 20 mg lisinopril, and health supplements totaling 1200 mcg vitamin A, 2.8 mg vitamin B1, 1.7 mg vitamin B2, 4.3 mg vitamin B6, 1200 mcg vitamin B9, 2.4 mcg vitamin B12, 120 mg vitamin C, 35 mcg vitamin D, 14 mg vitamin E, 200 mg calcium, 28 mg iron, 20 mg niacin, and 25 mg zinc. She took an additional 1000 mcg of vitamin B12 orally every other day and started taking 30 mg of apremilast twice daily approximately one month prior to this admission. Her past surgical history was remarkable for gastric sleeve surgery eight years ago.

**Figure 1:**
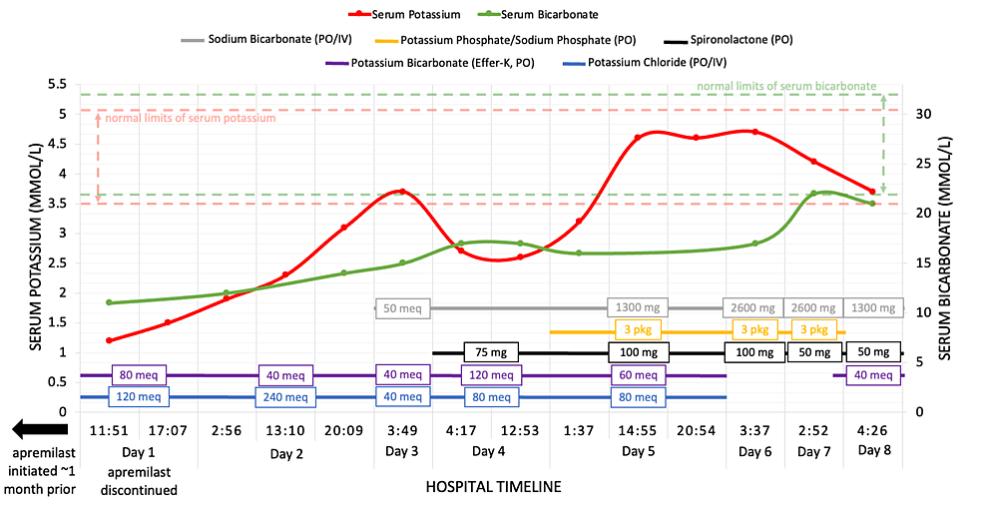
Illustration of the patient's serum potassium and bicarbonate levels during her hospitalization and the corresponding medical therapy utilized IV: Intravenous

An initial physical exam revealed a woman who appeared older than her stated age, frail, and cachectic. The oral cavity had white lesions compatible with thrush. Auscultation revealed a previously known systolic ejection murmur and poor airflow with coarse breath sounds. The abdominal exam was remarkable for a scaphoid abdomen, with hypoactive bowel sounds. Muscle strength was diffusely diminished, but no focal weakness was noted. Initial vital signs were oxygen saturation of 100% on room air, blood pressure 142/81 mmHg, temperature 36.9°C (98.5°F), heart rate 49/min, and respirations 18/min. All initial lab values are given in Table 1 and were remarkable for potassium 1.2 mmol/l, chloride 118 mmol/L, bicarbonate 11 mmol/L, magnesium 2.9 mg/dl, and a corrected calcium of 13 mg/dl. She was started on aggressive intravenous (IV) hydration, and on both IV and oral potassium repletion (see Figure 1). For her leukocytosis (20.4 K/mm^3^), she was empirically started on a seven-day course of 2 g ceftriaxone daily, in addition to nystatin therapy. Her home hydrochlorothiazide, lisinopril, over-the-counter supplements, and apremilast were discontinued. The unexpectedly high serum lipase level was noted to be down trending on hospital day two to 4838 units/L. The patient denied abdominal pain throughout hospitalization. Further workup consisted of a CT chest, abdomen, and pelvis, and an MRI brain given her cachectic appearance, hypercalcemia, and severe electrolyte abnormalities. Imaging revealed no lesions of the bones, adrenals, pancreas, or pituitary gland, and failed to identify a source of infection (see appendices). Given the severe leukocytosis, we investigated the possibility of hematologic malignancy. Peripheral blood smear revealed relative granulocytosis with marker patterns compatible with reactive features, thrombocytosis, increased CD4-to-CD8 ratio with no aberrant T-cell marker loss detected, no clonal B-cell populations, and no evidence of acute leukemia. Hypercalcemia workup returned an appropriately low parathyroid hormone (PTH), an undetectable PTH-related peptide, and normal levels of vitamin D. Her morning cortisol was elevated at >61.6 mcg/dL, while aldosterone and renin returned <1 ng/dL and <0.167 ng/mL/hr, respectively. Initially, it proved challenging to bring her potassium levels within normal limits despite aggressive supplementation (see Figure 1). Due to continued acidemia, we supplemented her with 50 mEq of sodium bicarbonate on day three. At this time, urine testing revealed elevated random urine potassium of 30 mmol/L, urine sodium of 31 mmol/L, urine chloride of 42 mmol/L, and urine pH of 6.5, indicative of potassium and bicarbonate wasting.

**Table 1:** Comparison of the patient’s initial versus final laboratory values Bolded values indicate the result is outside of the normal reference range for the respective parameter. N/A: Not applicable; eGFR: Estimated glomerular filtration rate; TSH: Thyroid stimulating hormone; Pro-BNP: Pro-B-type natriuretic peptide; PTH: Parathyroid hormone; PTH-rp: Parathyroid hormone-related protein; Initial: Lab value at admission; Final: Last known value prior to discharge

Laboratory component	Initial	Final	Normal range
White blood cell count	20.4 K/mm^3^	7.5 K/mm^3^	3.7-10.1 K/mm^3^
Red blood cell count	4.49 M/mm^3^	3.37 M/mm^3^	3.78-5.10 M/mm^3^
Hemoglobin	12.3 gm/dL	9.6 gm/dL	11.6-15.4 gm/dL
Hematocrit	37.4%	28.9%	34.9-44.1%
Mean corpuscular volume	83.4 fL	85.9 fL	79.2-97.2 fL
Platelet count	512 K/mm^3^	279 K/mm^3^	156-352 K/mm^3^
Serum sodium	141 mmol/L	136 mmol/L	137-145 mmol/L
Corrected serum sodium	142 mmol/L	136 mmol/L	135-146 mmol/L
Serum potassium	1.2 mmol/L	3.7 mmol/L	3.5-5.1 mmol/L
Serum chloride	118 mmol/L	109 mmol/L	96-107 mmol/L
Serum bicarbonate	11 mmol/L	21 mmol/L	22-32 mmol/L
Serum anion gap	12.0 mmol/L	6.0 mmol/L	3.0-11.0 mmol/L
Serum blood urea nitrogen	41 mg/dL	17 mg/dL	7 -20 mg/dL
Serum creatinine	1.30 mg/dL	0.70 mg/dL	0.7-1.5 mg/dL
eGFR	40 ml/min/1.73 m^2^	91 ml/min/1.73 m^2^	>60 ml/min/1.73 m^2^
Serum calcium	12.7 mg/dL	10.0 mg/dL	8.4-10.2 mg/dL
Corrected serum calcium	13.0 mg/dL	11.0 mg/dL	8.4-10.2 mg/dL
TSH	0.13 uIU/ml	N/A	0.464-4.68 uIU/ml
Albumin	3.6 gm/dL	N/A	3.5-5.0 gm/dL
Creatine kinase	281 units/L	N/A	30-170 units/L
Pro-BNP	1770 pg/ml	N/A	0-299 pg/ml
Troponin	0.101 ng/ml	N/A	0-0.034 ng/ml
Lipase	>8000 units/L	N/A	23-300 units/L
Ceruloplasmin	21.7 mg/dl	N/A	19.0-39.0 mg/dl
Vitamin B9	>20 ng/ml	N/A	2.76- 20 ng/ml
Vitamin B12	>1000 pg/mL	N/A	239-931 pg/ML
1,25 vitamin D	74.1 pg/ml	N/A	24.8-81.5 pg/mL
25 vitamin D	97.3 ng/ml	N/A	30-100 ng/ml
PTH	13.6 pg/ml	N/A	18.5-88.0 pg/mL
PTH-rp	undetectable	N/A	<2.5 pmol/L
Aldosterone	<1 ng/dl	N/A	7-30 ng/dl
Renin	<0.167 ng/ml/hr	N/A	0.167-5.380 ng/ml/hr
Cortisol	>61.6 mcg/dl	N/A	5-25 mcg/dl

Given the degree of electrolyte abnormalities, nephrology was consulted on day four and the diagnosis of distal RTA was presumed. She was started on spironolactone, and sodium bicarbonate, which resulted in a dramatic improvement in her potassium on hospital day five. By hospital day seven, her bicarbonate levels had improved to 22 mmol/L and her potassium had increased to 3.7 mmol/L without the use of IV potassium supplementation. In addition, she started eating, returned to her baseline mental status, and was capable of performing all activities of daily living. She was discharged with new prescriptions for sodium bicarbonate 1300 mg twice daily, and 50 mg spironolactone daily and instructed to continue her home medications except for her vitamin D supplements and hydrochlorothiazide.

We were unable to schedule an in-office appointment to assess the patient, however, on telephone follow-up 53 days following discharge, the patient reported continued improvement with good appetite and regained strength. Her follow-up blood work has remained without abnormalities (except for continued anemia). She continues to take her sodium bicarbonate and spironolactone as prescribed. Since discharge, she attempted to restart her apremilast but reported generalized weakness, lethargy, and a feeling of being overall unwell. She subsequently decided to discontinue the agent indefinitely.

## Discussion

The case illustrates a complex interplay of conditions in a 73-year-old female with psoriatic arthritis, presenting with severe electrolyte imbalances indicative of type 1 RTA. Initial interventions focused on correcting these derangements while exploring potential underlying causes. The comprehensive diagnostic approach ruled out common culprits like multiple myeloma, hyperaldosteronism, pituitary adenoma, hyperparathyroidism, and malignancy.

Hypercalcemia was attributed to severe dehydration rather than primary hyperparathyroidism or malignancy, given the absence of elevated PTH, normal vitamin D levels, a normal bone marrow biopsy, and no evidence of malignancy on imaging. Additionally, hematologic malignancy was excluded with a recent bone marrow biopsy and peripheral blood smear during her hospitalization that showed absolute granulocytosis without evidence of malignancy. Peripheral blood smear also ruled out other common culprits of hypercalcemia, such as multiple myeloma. Suspicions of adrenal or pituitary involvement were dismissed due to suppressed aldosterone and renin levels and the absence of Cushingoid features. This was in alignment with endocrinology’s recommendations that her elevated morning cortisol was likely caused by a physiologic stress response secondary to starvation and dehydration. Ultimately, evidence of urinary potassium and bicarbonate wasting led to the diagnosis of type 1 RTA, successfully managed with spironolactone and sodium bicarbonate.

Despite our comprehensive workup, unanswered questions persist. The contribution of psoriatic arthritis cannot be overlooked given the systemic nature of the disease and its known potential to cause organ dysfunction. The cause of the elevated lipase levels remains unclear. Our patient’s physical examination lacked substantiated clinical evidence consistent with acute pancreatitis. Additionally, the abdominal CT scans showed a pancreas that was normal in size with no enhancing lesions or peripancreatic inflammation with a normal caliber pancreatic duct, furthering the contention that the elevation in serum lipase may be due to alternative causes other than acute or chronic pancreatitis. While other causes, including renal impairment and autoimmune diseases, have been postulated as alternative causes [9], elevations of this caliber secondary to alternative causes are seldom reported in the literature. Psoriasis has been associated with chronic pancreatitis [10]. However, this is unlikely in our patient given the significantly elevated serum lipase levels and lack of characteristic pancreatic scarring and fibrosis that would be expected on abdominal CT. Additionally, an analysis of 15 randomized, placebo-controlled studies involving apremilast showed no association with acute pancreatitis [11].

It is accepted that various autoimmune conditions may contribute to the development of clinically significant distal RTA [5], although psoriatic arthritis itself lacks substantial evidence. Our patient reported prior use of secukinumab since she was first diagnosed with psoriatic arthritis. However, she was transitioned to apremilast one month prior to her admission. Despite its established utility in treating psoriatic arthritis, its association with distal RTA remains uncharted territory [11]. One prior case report provided strong evidence that apremilast may have been the cause of a similar clinical presentation [7]. The temporal association between apremilast initiation, clinical presentation, and adverse reactions post-discharge suggests a potential link, highlighting the need for in-depth investigation. Based upon the Naranjo Adverse Drug Reactions Probability Scale, the probability of the drug as the cause of our patient’s presentation is "possible." The temporal association and case report by Perrone et al. are noteworthy, however, we cannot definitively exclude other etiologies for this patient’s presentation such as psoriasis.

This case emphasizes the importance of continued research into less common etiologies of RTA, especially in the context of autoimmune conditions and medication use. Ascertaining the adverse effects profile of apremilast and its possible role in drug-induced RTA should be a focus of future investigations. The presented case urges clinicians and researchers to remain vigilant for unexpected associations and contribute to the evolving understanding of complex clinical presentations, enabling more informed patient care.

## Conclusions

The presented case highlights the diagnostic challenges encountered in a 73-year-old patient with psoriatic arthritis who developed severe electrolyte imbalances indicative of distal RTA. The successful management of the patient's condition with spironolactone, sodium bicarbonate, and rehydration emphasizes the importance of a comprehensive diagnostic approach in complex clinical scenarios. The temporal association between the initiation of apremilast and the clinical presentation raises concerns about the medication's potential role in drug-induced distal RTA, accentuating the need for continued research into less common etiologies of distal RTA, particularly in the context of autoimmune conditions and medication use. This case serves as a call for clinicians and researchers to remain vigilant for unexpected associations, contributing to the evolving understanding of complex clinical presentations and guiding future investigations into the adverse effects of apremilast.

## Figures and Tables

**Figure 2:**
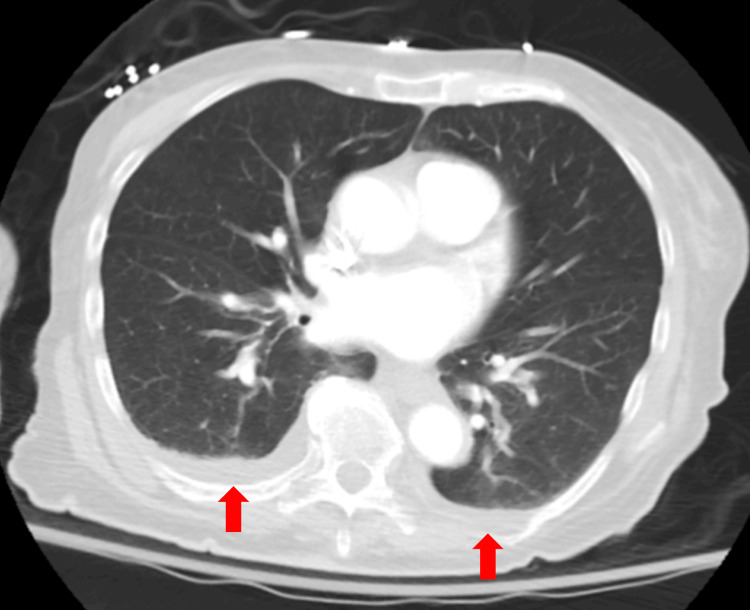
CT chest with contrast in the axial plane revealed small bilateral pleural effusions without other acute intrathoracic abnormalities CT: Computed tomography

**Figure 3:**
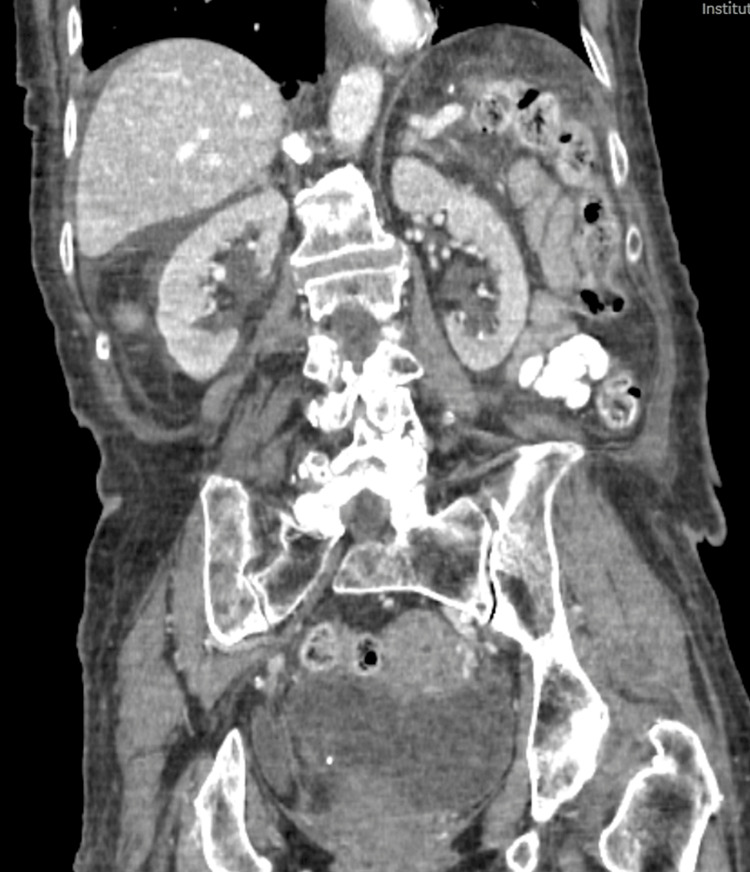
CT abdomen and pelvis with contrast in the coronal plane revealed no lesions or hyperplasia of the adrenals and no evidence of acute intra-abdominal or pelvic abnormalities CT: Computed tomography

**Figure 4:**
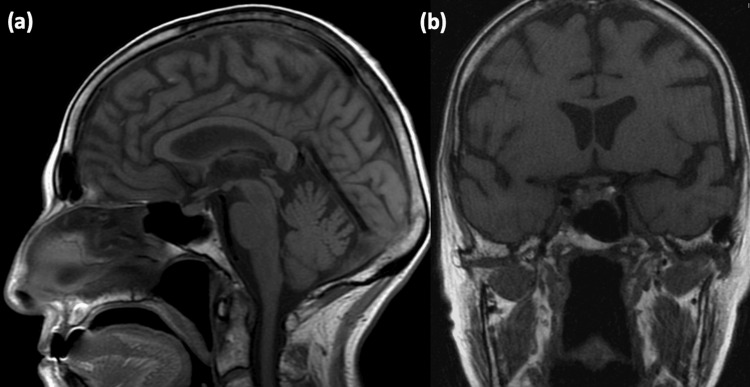
T1 weighted MRI brain in the sagittal (a) and coronal (b) planes revealed no mass, acute infarct, hemorrhage, or edema MRI: Magnetic resonance imaging
